# The synergistic antitumor effect of Huaier combined with 5-Florouracil in human cholangiocarcinoma cells

**DOI:** 10.1186/s12906-019-2614-5

**Published:** 2019-08-07

**Authors:** Zhaoyu Fu, Kexin Ma, Bing Dong, Chongyu Zhao, Chi Che, Chengyong Dong, Rixin Zhang, Haibo Wang, Xiang Wang, Rui Liang

**Affiliations:** 1 Division of Hepatobiliary and Pancreatic Surgery, Department of General Surgery, The Second Affiliated Hospital of Dalian Medical University, 467 Zhongshan Road, Dalian, 116023 Liaoning China; 2 Institute of Cancer Stem Cell, Dalian Medical University, Dalian, Liaoning China

**Keywords:** Cholangiocarcinoma, Huaier, 5-Florouracil, Synergistic effect

## Abstract

**Background:**

5-Florouracil (5-FU) is a commonly used chemotherapeutic drug for cholangiocarcinoma, whereas it has unsatisfactory effect, and patients often have chemo-resistance to it. The combination of chemotherapeutic agents and traditional Chinese medicine has already exhibited a promising application in oncotherapy. Huaier extract (Huaier) has been used in clinical practice widely, exhibiting good anti-tumor effect. This paper aims to investigate the possibility of combination 5-FU and Huaier as a treatment for cholangiocarcinoma.

**Methods:**

A series of experiments were performed on the Huh28 cells in vitro, which involved cell proliferation, colony formation, apoptosis, cell cycle, migratory and invasive tests. Besides, western blots were also performed to examine the potential mechanism of 5-FU.

**Results:**

The combination effect (antagonism, synergy or additive) was assessed using Chou-Talalay method. Using the CCK-8 and Colony formation assay, the anti-proliferation effect of 5-FU combined with Huaier was observed. Apoptosis inducing and cell cycle arrest effect of the combination of two drugs were assessed by flow cytometry. To determine the combined treatment on cell immigration and invasion ability, wound healing and Transwell assay were performed. The above experiment results suggest that the combined 5-FU and Huaier, compared with treatment using either drug alone, exhibited stronger effects in anti-proliferation, cycle arrest, apoptosis-induced and anti-metastasis. Further, western blot results reveal that the inhibition of STAT3 and its target genes (e.g. Ki67, Cyclin D1, Bcl-2 and MMP-2) might be set as the potential therapeutic targets. Besides, the inhibition of combination treatment in proteins expression associated with proliferation, apoptosis, cell cycle and metastasis was consistent with that of previous phenotypic experiments.

**Conclusions:**

Huaier combined with 5-FU exhibited a synergistic anti-tumor effect in Huh28 cell. Furthermore, the mechanisms might be associated with the activation and translocation of STAT3, as well as its downstream genes.

**Electronic supplementary material:**

The online version of this article (10.1186/s12906-019-2614-5) contains supplementary material, which is available to authorized users.

## Background

Cholangiocarcinoma (CCA) is considered as the second most commonly-used primary hepatic tumor [1]. CCA can be located deep in the liver and concealed anatomically, thereby rendering treatment and early diagnosis extremely difficult to achieve [2]. The rates of survival vary with the anatomic location of the carcinoma and the extent of metastasis [3]. Patients diagnosed with a intrahepatic, distal extrahepatic and hilar CCA receiving surgical intervention have the five-year survival rates of 22–44%, 27–37%, and 11–41%, respectively [4]. Thus, the treatment options for CCA patients are limited. Various drugs have been used for treating CCA patients, the most common of which is 5-fluorouracil, a chemotherapeutic drug used in digestive system tumors widely for its low cost. Moreover, gemcitabine (GEM), cisplatin (CIS) and doxorubicin (DOX) have had wide applications as well [5]. However, CCAs have a poor response to these currently available chemotherapeutic agents [6]. In clinical practice, physicians have attempted to combine different chemotherapy drugs, including 5-FU combined with cisplatin, BAF (bleomycetin + adriamycin + 5-FU) and FAM (5-FU + adriamycin + mitomycin) [7], to maximize the anti-cancer effect, whereas the effects of the combined drugs are not all ideal.

Thus, new treatments or different drug combinations should be found. Recently, traditional Chinese medicine (TCM), with a long history in the treatment of different diseases in China, has aroused increasing international recognition [8]. In the field of anti-cancer treatment, TCM is popular for relatively mild adverse effects because it preferentially kills cancer cells and inhibits metastasis compared with western medicine and provides more options for clinical practice [9]. In the United States and Europe, several valuable TCMs have been adopted as alternative or complementary medicines.

Huaier is a fungus that has been used in traditional Chinese medicine for more than 1000 years. Proteoglycans, consisting of polysaccharides, amino acids and water, are identified as the major components of Huaier extract [10]. In recent years, Huaier has been reported exhibiting a wide range of anti-cancer functions, including the induction of apoptosis and anti-angiogenesis, without obvious side effects. Numerous clinical applications suggested that Huaier has satisfactory therapeutic effects in the treatment of solid malignancies (e.g. liver cancer [11], gastric cancer [12], cervical cancer [13], breast cancer [14], and lung cancer [15]). Besides, the combined treatment of Huaier and Chemotherapy drugs, (e.g. paclitaxel [16] and cisplatin [17]) has been proposed to enhance efficacy of treatment and reduce toxic effects. While, whether Huaier alone or in combination with other drugs can become a potential treatment for cholangiocarcinoma remains unclear.

Signal transducer and activator of transcription 3 (STAT3) is considered an oncogene, being continuously activated in more than 50% of human solid tumor (e.g. Cholangiocarcinoma) [18]. At the point when phosphorylated at tyrosine 705 (Tyr705), STAT3 will be dissociated from the receptor and translocated from cytosol to the nucleus in which it regulates target genes expression correlated with proliferation, cell cycle, apoptosis and carcinogenesis [19]. Serval pro-proliferative, anti-apoptotic and pro-metastasis genes (e.g. cyclin-D1 Bcl-2 and MMP-2 [20]) have been reported to be associated with STAT3 activation and translocation. Thus, STAT3/p-stat3 may be a potential target of Cholangiocarcinoma treatment.

In this study, we aimed to investigate the synergistic anti-cholangiocarcinoma effect of two drugs in vitro (the chemotherapy drug fluorouracil and traditional Chinese medicine Huaier). We focused on the regulation of tumor proliferation, apoptosis, migration and invasion by treatment of either agent alone or both of them. Our results indicated that the combination of 5-FU and Huaier is a method effectively treating human cholangiocarcinoma Huh28 cells. Its potential molecular mechanism is the inhibition of the phosphorylation of STAT3(Tyr705), and its target genes include cyclin-D1 Bcl-2 and MMP-2.

## Methods

### Cell culture

The cholangiocarcinoma cell line Huh28 was provided by the Japanese Collection of Research Bioresources Cell Bank (Osaka, Japan), and all cells were grown in 1640 medium (RPMI-1640) containing 10% fetal bovine serum (FBS) at 37 °C in a 5% CO2 atmosphere.

### Agents and antibodies

Huaier extract was kindly provided by Qidong Gaitianli Pharmaceutical Co., Ltd. (Jiangsu, China). Huaier extract was dissolved in RPMI-1640 medium to produce 100 mg/ml stock solution. Subsequently, the stock solution was sterilized with a 0.22-μm filter and then stored at 4 °C. 5-FU was purchased from Sigma (St. Louis, MO, USA) and then dissolved in RPMI-1640 medium before use. IL-6/ Interleukin-6 was provided by Sino Biological Inc. (Beijing, China). The antibodies of Ki67, PCNA, Mcl-1, Bcl-2, Cyclin A2, CDK2, N-cadherin, Vimentin, MMP-2, MMP-9, GAPDH and Lamin B1 were purchased from Abcam (Cambridge, UK). The antibodies of STAT3 and p-STAT3 were provided by Cell Signaling Technology (Beverly, MA, USA).

### Cell viability assay

Cell growth and viability were detected using CCK-8 assay. Huh28 cells was cultured overnight at 5 × 10^3^ cells/well in 96-well plates. Subsequently, the original medium was replaced by a complete medium containing Huaier, 5-FU or their combination in different concentrations. After 24 h or 48 h of incubation, CCK-8 was added to each well, and then the plates were further incubated at 37 °C for 2 h. Next, the optical density (OD) values of samples were measured with an EnSpire plate reader (PerkinElmer) at 450 nm. The cells viability was calculated by the following equation: viability = (the OD values of treatment groups/the OD values of control group) × 100%.

### Drug combination therapy effect evaluation

Using Chou and Talalay method (1984), the drug-drug interaction between Huaier and 5-FU was investigated, and the result of combination index (CI) values and dose reduction index (DRI) values were calculated using CompuSyn software.

### Colony formation assay

Huh28 cells were incubated at 1 × 103 cells/well in 6-well plates and then underwent different treatments for 48 h. Subsequently, previous medium was replaced by complete medium for further 10 days’ incubation. The colonies were stained with 1% crystal violet for 10 min and then washed with PBS before use. After drying, the visible colonies were photographed and then counted.

### Apoptosis assay

After undergoing the treatment of huaier, 5-FU or combination therapy for 48 h, the Huh 28 cells were trypsinized with trypsin without EDTA. After cells were washed and centrifugated at 1000 rpm/5 min with cold PBS twice, at least 1 × 105/ml cells had to be collected and resuspended in buffer. Subsequently, using an Annexin V-FITC/PI-staining kit (KenGen Biotechnology Co., Nanjing, China), cells were stained with 5 μL Annexin V-FITC and 5 μL propidium iodide (PI) sequentially. The percentage of apoptotic cells was quantified using C6 flow cytometer (Becton Dickinson and Co., Franklin Lakes, New Jersey, USA).

### Cell cycle analysis

After undergoing the treatment of huaier, 5-FU or combination therapy for 48 h, cells were trypsinized, washed and resuspended in cold methanol to make into single cell suspensions and stored at 4 °C overnight. Subsequently, using Cell cycle staining Kit (KenGen Biotechnology Co., Nanjing, China), at least 1 × 106/ml cells were resuspended in staining buffer premixed by 50 μl RNase A and 450 μl propidium iodide (PI). After being incubated for 30 min, the samples were also examined by C6 flow cytometer, and the results were analyzed using Flowjo software.

### Wound healing assay

Huh28 cells were cultured in 6-well plates in advance. When the cells grew to cover 80–90% of the plate bottom, and the detached cells were washed with PBS, an artificial wound was made using a 100 μL pipette tip. Cells were exposed to Huaier and 5-FU alone or in combination for 48 h. Afterwards, cells migration was observed and the wound was photographed using a microscope equipped with digital camera (Leica Microsystems, Germany). The wound and repaired area were measured with image J software.

### Cell migration and invasion assay

Cell migration assay was performed using Transwell chambers with 8.0-μm pore size membrane. Cells were treated with Huaier and 5-FU alone or in combination for 48 h and collected in advance. Subsequently, 250 μL 1 × 105 cells in 250 μL medium without FBS were added to upper chamber, while 500 μL medium with 10% FBS was added to the lower chamber. After being incubated for 48 h, cells on the upper surface were wiped away, and those on the lower surface were stained with 0.1% crystal violet. Lastly, photomicrographs were taken, and the number of migrated cells was calculated in three random microscope fields. For cell invasion assays were performed following the procedures consistent with the assay of cell migration, except for the step that upper surface of Transwell chamber was coated with Matrigel (2.5 mg/L).

### Western blot analysis

Cells were pre-treated with Huaier and 5-FU alone or in combination for 48 h in advance. The total protein extraction and cytoplasmic/nuclear protein isolation were performed as previously described [21]. Protein concentrations were measured using BCA protein assay. The protein samples were premixed with 5 × loading buffer, separated and then transferred to PVDF membranes in 10% SDS-PAGE. Subsequently, the membranes were incubated with specific primary antibodies and appropriate secondary antibodies. Lastly, the bands were visualized using an enhanced chemiluminescence.

### Statistical analysis

SPSS 17.0 and GraphPad Prism 7 software were used for the statistical analysis and the data presentation. Comparisons among different groups were drawn using Student’s t-tests and one-way ANOVAs. Data are expressed as the mean ± standard deviation of three repeated experiments. A level of *P* < 0.05 was considerate as statistically significant.

## Result

### The inhibiting effects of Huaier and 5-FU alone or in-combination on Huh28 cells and the synergistic anti-proliferative effects of two drugs

**Fig. 1:**
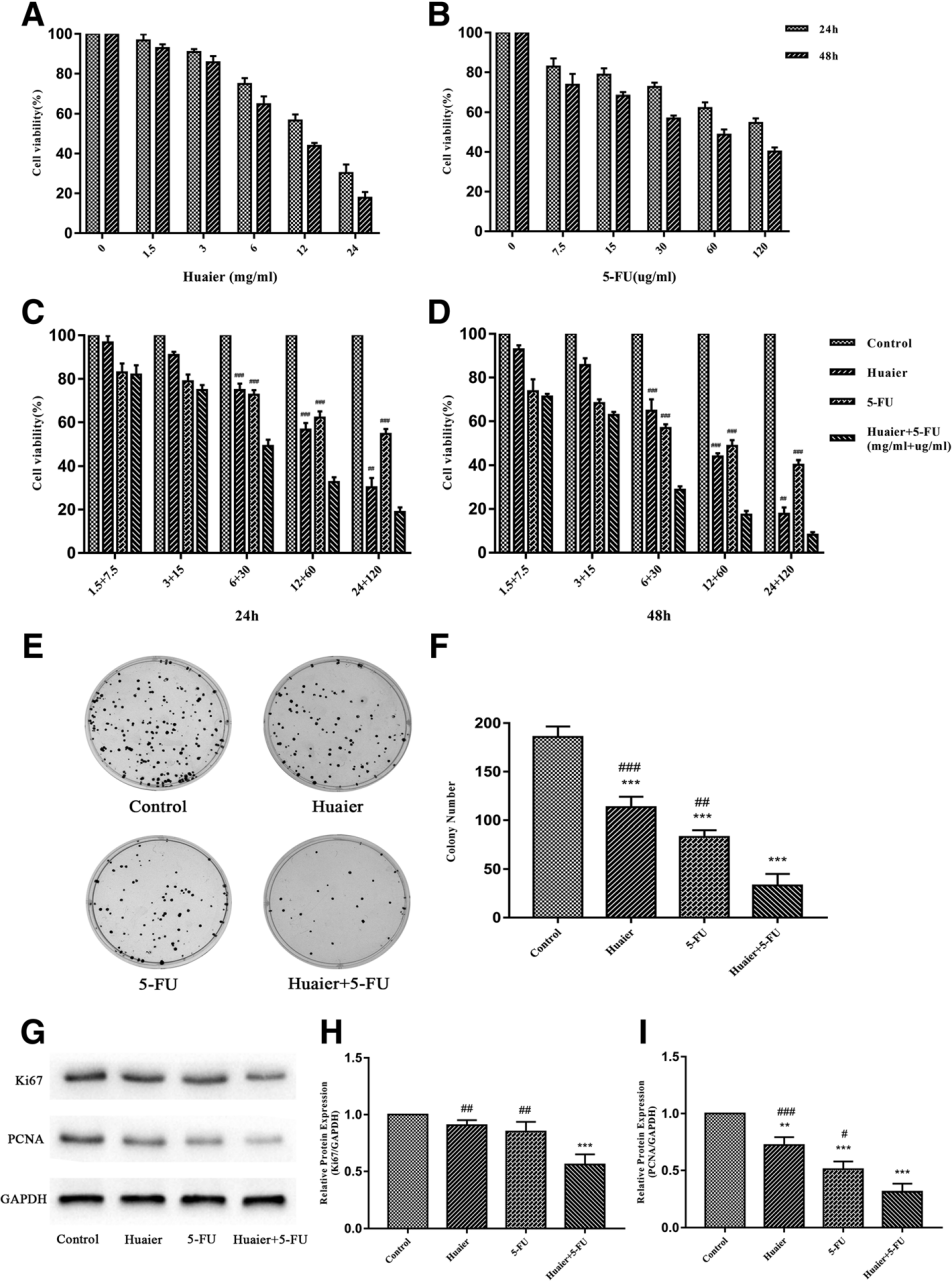
The effect of Huaier and 5-FU treated alone or in Combination on cell proliferation and colony formation in Huh28 cells. **a** and **b** After being treated with Huaier (1.5-24 mg/ml) or 5-FU alone (7.5-120 μg/ml) in different concentrations for 24 h and 48 h, the cell viability was measured with CCK-8 assay. **c** and **d** Cell proliferation ability was also assessed after cells were treated with Huaier and 5-FU alone or in combination for 24 h and 48 h. **e** and **f** The inhibiting effects on colony formation by treated monotherapy or in combination were shown, and the colony numbers were calculated. **g** and **h** The protein expressions of Ki67 and PCNA in Huh28 cells after 48 h treatment with Huaier and 5-FU alone or in combination were measured with western blot, and relative optical density of the proteins expression was analyzed using Image J. All data are expressed as the mean ± S.D. of three separate experiments. ^*^P < 0.05, ^**^P < 0.01, ^***^P < 0.001, compared with control group, while ^#^*P* < 0.05, ^##^P < 0.01, ^###^*P* < 0.001, compared with combination group. **i** The panel labels “24h” and “48” represent the samples were detected after treatment 24 hours and 48 hours respectively. The panel labels “Control”, “Huaier”, “5-FU” and “Huaier+5-FU” represent the samples were treated by neither of two drugs, Huaier extract, 5-Fluorouracil and combination of two drugs respectively

To assess the effects of Huaier or 5-FU as single-agent therapies on cell viability of Huh28 cells, CCk-8 assay was performed. Both Huaier and 5-FU treatment showed a concentration-dependent and time-dependent inhibiting effect in cell viability. It was suggested that the proliferation inhibition rate of Huaier (1.5-24 mg/ml) were 3.3–69.8% and 7.1–82.2% respectively after Huh28 cells were treated for 24 and 48 h (Fig. 1a), while the proliferation inhibition rate of 5-FU (7.5–120 mg/ml) was 17.1–45.4% and 26.3–59.8% after Huh28 cells were treated for 24 and 48 h, respectively (Fig. 1b). The cytotoxicity of Huaier was slight at low concentration (7.5 and 15 μg/ml), whereas the inhibiting effect was significantly up-regulated with the rise in concentration (6 mg/ml or higher concentration). However, unlike Huaier, the cytotoxicity of 5-FU on Huh28 cells increased gradually with the rise in the concentration of 5-FU. The survival rate of Huh28 cells treated with Huaier was close to 30% (24 mg/ml,24 h), while it remained above 50% of survival rate under the high concentration treatment of 5-FU (120 μg/ml, 24 h).

To determine whether the antitumor effects were enhanced by the combination of Huaier and 5-FU, 1: 5(Huaier: 5-FU) ratio for combination was investigated in Huh28 cells. Compared with the inhibition rate of the proliferation of Huh28 cells treated with Huaier or 5-FU alone for 24 h or 48 h, Huaier in combination with 5-FU significantly increased Huh28 cells proliferation inhibiting effect **(**Fig. 1c and d**)**. Subsequently, Chou-Talalay method was used to calculate the combination index (CI) and the dose-reduction index (DRI) to further verify the effect of the combination. The results are listed in **(**Tables 1 and 2**)** and **(**Additional file 1**:** Table S1 and S2**)**. When the concentration of Huaier combined 5-FU was 6 ml/mg, 30 μg/ml or higher, the interaction between two drugs was synergistic. Besides, the combination of two drugs could reduce the dosage of either drug to achieve the same inhibiting effect. After the treatment for 24 h and 48 h, the lowest combination index was reached when the concentration of Huaier combined 5-FU was 6 ml/mg and 30 μg/ml, respectively. For the above reasons, Huaier (6 ml/mg) and 5-FU (30 μg/ml) monotherapy or combination therapy were selected for further treatments in this study.

**Table 1:** The fraction affected level (Fa) of Huaier and 5-FU in individual or in combination, and the combination index (CI) values of combination after 24 h

Huaier (mg/ml)	5-FU (μg/ml)	Concentration ratio (Hauier: 5-FU)	Fa of Huaier	Fa of 5-FU	Fa of combination	CI
1.5	7.5		3.30%	17.10%	18.10%	1.09
3	15		9.10%	21.20%	25.20%	1.16
6	30	1:05	25.20%	27.40%	50.90%	0.59
12	60		43.40%	37.90%	67.40%	0.63
24	120		69.80%	45.40%	81.20%	0.71

CI was used for a quantitative measure of the extent of the interaction between two drugs. The CI > 1 indicates antagonism; CI < 1 means synergy; CI = 1 indicates additive effects. The CI values< 1 indicates that the combination (24 h) of Huaier (1.5–24 mg/ml) and 5-FU (7.5–120 μg/ml) was synergistic on Huh28 cells

**Table 2:** The dose of Huaier or 5-FU alone to achieve the same fraction affected level of in-combination and the dose reduction index (DRI) values for Huaier combined with 5-FU after 24 h

Fa of combination	Dose of Huaier (mg/ml)	Dose of 5-FU (μg/ml)	DRI of Huaier	DRI of 5-FU
18.10%	5.02	9.46	3.34	1.26
25.20%	6.63	21.26	2.21	1.42
50.90%	13.96	184.34	2.33	6.14
67.40%	22.05	694.58	1.84	11.58
81.20%	35.91	2861.14	1.5	23.84

The DRI indicates the fold of the dose reduction of tested Huaier and 5-FU in-combination compared with that of each individual drug. To achieve a 50.9% of inhibiting effect in Huh28 cells proliferation when the CI was at the lowest (0.59, as listed in Table 1A) in combination after 24 h, the concentrations of Huaier and 5-FU were reduced 2.33~ folds and 6.14~folds in combination, while their individual dose reached to 13.96 mg/ml and 184.34 μg/ml

To investigate the combined treatment on the proliferation of Huh28 cells, colony formation assay was performed. As shown in **(**Fig. 1e and f**)**, the combined treatment with Huaier and 5-FU significantly reduced the formation of colony compared with that of the single drug treatment.

To further confirm the anti-proliferation effect of monotherapy or combination, western blot was performed to measure the protein expression levels of two Ki67 and PCNA which are closely associated with cellular proliferation. Compared with control and single agent groups, combination group significantly down-regulated protein expressions of Ki67 and PCNA **(**Fig. 1g and h**)**.

All these results suggest that combined Huaier and 5-FU had a significant anti-tumor synergistic effect.

### Huaier combined with 5-FU synergistically promoted cell apoptosis induction

**Fig. 2:**
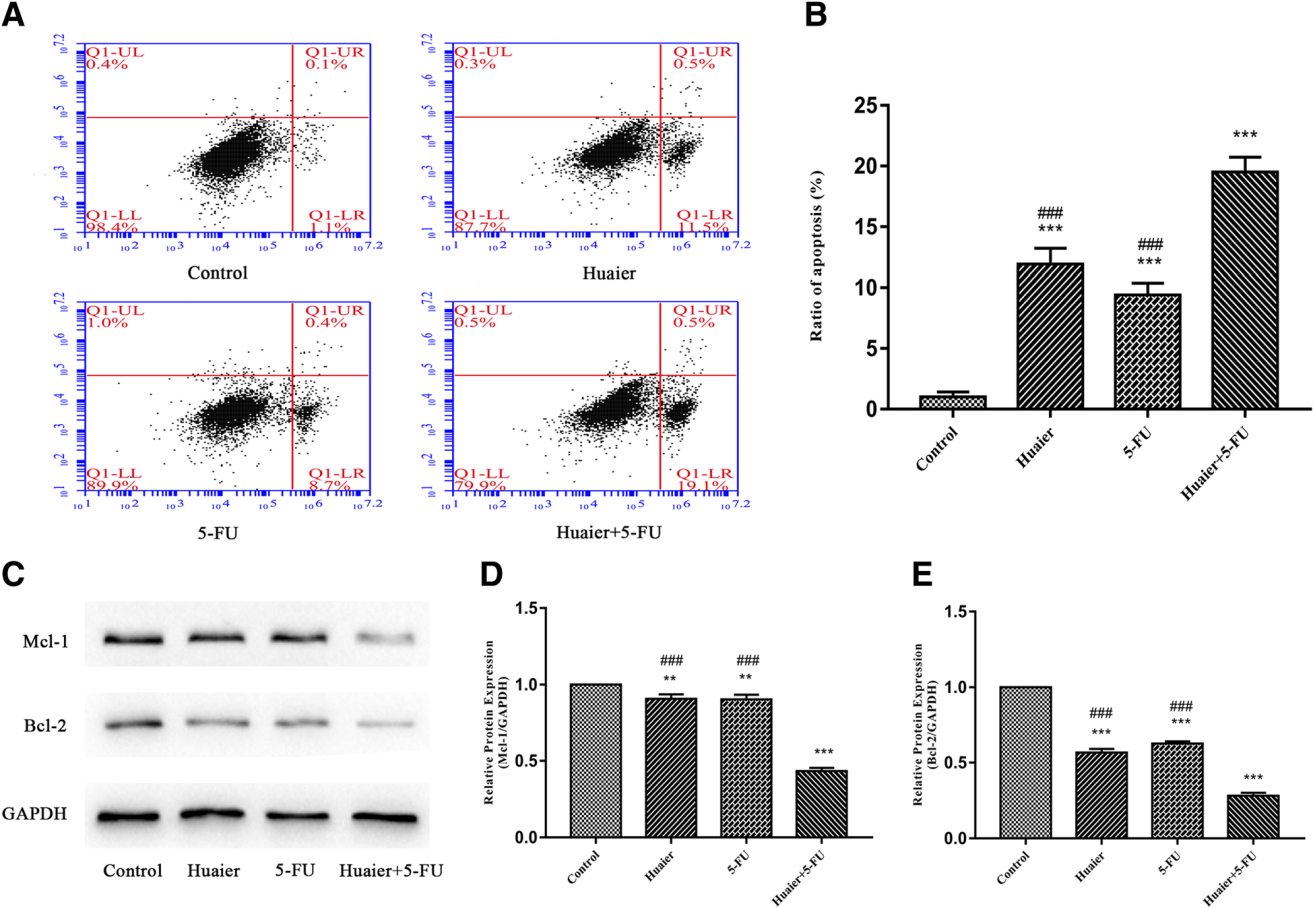
Apoptosis induction effect of Huaier and 5-FU in single agent or combination on Huh28 cells. **a** Flow cytometry histograms of Huh28 cells after 48 h treatment with Huaier (6 mg/ml) and 5-FU (30 μg/ml) alone or in combination, the samples were detected using AV/PI double-staining method. **b** The percentage of apoptotic cells was calculated, and results are shown in bar graph. **c**, **d** and **e The** expression levels of anti-apoptotic protein Mcl-1 and Bcl-2 in Huh28 cells after 48 h of different treatments were detected by Western blot, and the relative optical densities of proteins were analyzed by Image J. All results are expressed as mean ± SD of three independent experiments. *P < 0.05, **P < 0.01, ***P < 0.001, compared with control group, while ^#^*P* < 0.05, ^##^*P* < 0.01, ^###^*P* < 0.001, compared with combination group

The effect of apoptosis induction on Huh28 cells caused by monotherapy or combination treatment was examined using flow cytometry. Results suggest that the treatment with Huaier (6 mg/ml) or 5-FU (30 μg/ml) alone could result in apoptosis of Huh28 cells. As shown in **(**Fig. 2a and b**)**, the apoptosis induction effect of Huaier was a little stronger than that of 5-FU (12.0% vs 9.1%, *p*>0.05). When Huaier (6 mg/ml) was combined with 5-FU (30 μg/ml), the treatment exhibited a more significant apoptosis induction on Huh28 cells than any other agent (19.6%, *p* < 0.001).

To gain insights into the underlying mechanism of apoptosis induction effect caused by Huaier (6 mg/ml), 5-FU (30 μg/ml), and their combination, we detected the expressions of two anti-apoptotic protein Mcl-1 and Bcl-2, which were considered a therapeutic target in tumor apoptosis process. The result indicates that the expressions of Mcl-1 and Bcl-2 were obviously down-regulated by combination treatment in Huh28 cells, compared with those in control or single agent groups **(**Fig. 2c, d and e**)**.

### Combination therapy of Huaier with 5-FU induced cell cycle arrest in Huh28 cells

**Fig. 3:**
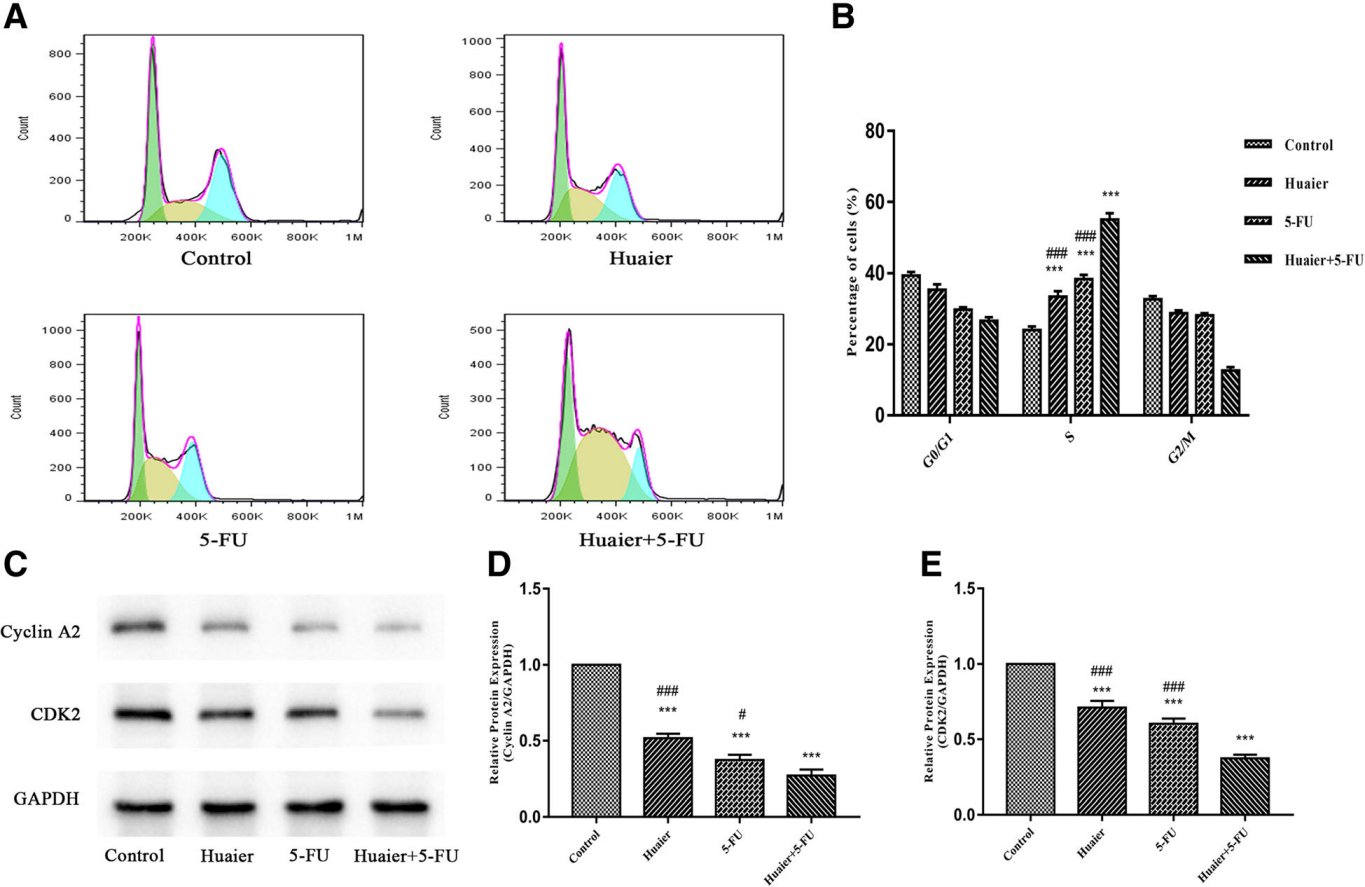
cell cycle arrest effect of Huaier and 5-FU in single agent or combination on Huh28 cells. **a** DNA content-based cell cycle of Huh28 cells after 48 h Huaier and 5-FU alone or in combination treatment were analyzed using Flow cytometry. **b** Percentage of cells in S phases. **c**, **d** and **e** The expression variations of cell cycle related proteins under different treatment conditions were detected using Western blot, and relative optical densities of proteins were analyzed by Image J. All results are expressed as mean ± SD of three independent experiments. ^*^P < 0.05, ^**^P < 0.01, ^***^P < 0.001, compared with control group, while ^#^*P* < 0.05, ^##^*P* < 0.01, ^###^*P* < 0.001, compared with combination group

To determine whether the treatment of Huaier and 5-FU alone or in combination could affect cell cycle of Huh28 cells, flow cytometry analysis was conducted, and the percentage of cells in different phases was detected. The results are shown in (Fig. 3a and b). Compared with control group, the percentage of cells in S phase increased after being the cells were exposed to Huaier (from 23.8 to 33.3%) and 5-FU (from 23.8 to 38.3%) alone. Besides, the combination treatment led to a greater increase in the percentage compared with either agent (from 23.8 to 56.2%). Thus, combined Huaier with 5-FU led a synergistic cell cycle arrest in both S phases on Huh28 cells. To further investigate the cell cycle arrest induced by combination treatment, the western blot result of several proteins was analyzed, known as cell-cycle checkpoints, Cyclin A2 and CDK2 associated with S phase. As shown in (Fig. 3c), the expressions of these two types of cell-cycle related proteins were down-regulated. This indicated that S phases arrest were induced by Huaier and 5-FU in monotherapy or combination treatment, and the combination of Huaier with 5-FU could have a stronger effect on cell cycle arrest than either agent alone.

### Combination Huaier with 5-FU synergistically inhibited the motility of Huh28 cells

**Fig. 4:**
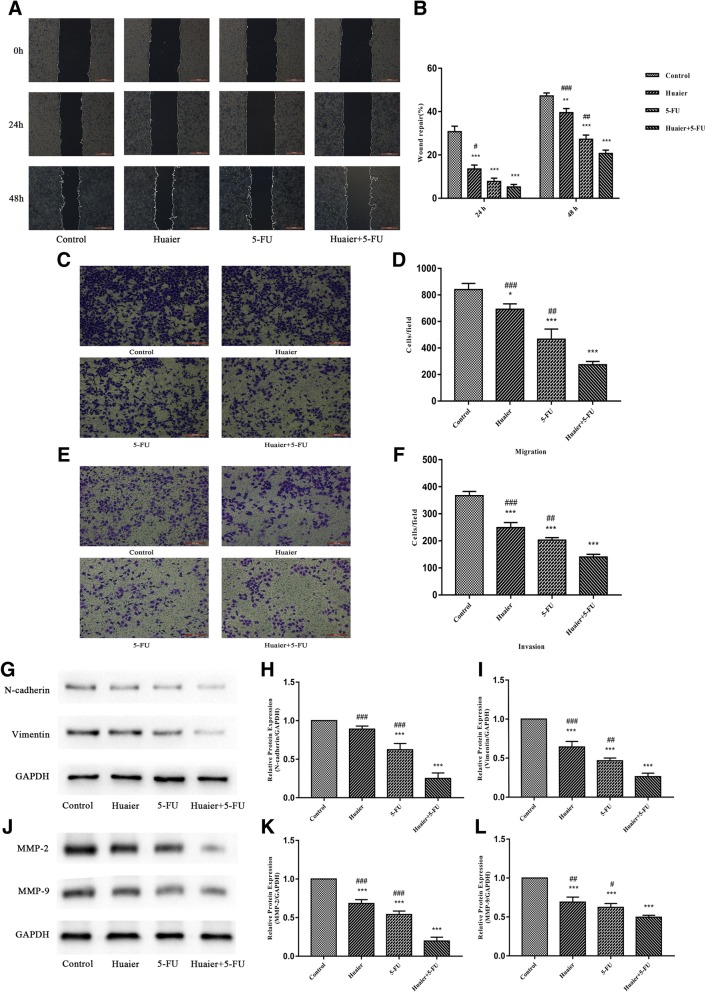
Effects of treatment with Huaier and 5-FU in single drug or combination on migratory and invasive abilities of Huh28 cells. **a** and **b** Representative image of wound healing assay of Huh28 cells at 0, 24 and 48 h after wound scratch when exposed to either single agent or combination treatment, The results of wound repair inhibition under different treatment conditions were compared. **c** and **d** Representative images of Transwell assay for migration using transwell chambers without Matrigel coating, Huh28 cells that had migrated through the filter after treatment with Huaier and 5-FU monotherapy or in combination for 48 h were calculated. **e** and **f** Representative images of Transwell assay for invasion using Transwell chambers with Matrigel coating, Huh28 cells that had penetrated through the filter after treatment with Huaier and 5-FU monotherapy or in combination for 48 h were calculated. **g**, **h**, **i**, **j**, **k** and **l** Western blot results of N-cadherin, Vimentin, MMP-2 and MMP-9 proteins expression after 48 h treatment with Huaier and 5-FU alone or in combination, and relative optical density of proteins were analyzed based on Image J. All results are expressed as mean ± SD of three independent experiments. ^*^P < 0.05, ^**^P < 0.01, ^***^P < 0.001, compared with control group, while ^#^*P* < 0.05, ^##^*P* < 0.01, ^###^*P* < 0.001, compared with combination group

To investigate whether Huaier and 5-FU alone or in combination treatment has an inhibiting effect on Huh28 cells migration and invasion, wound healing assays in vitro were performed as the first step. As shown in (Fig. 4a and b), wound repair in Huh28 cells after treatment with Huaier and 5-FU alone or in combination for 24 h or 48 h were delayed compared with control group. The repaired area percentage after 48 h exposure of Huaier and 5-FU alone were 39.5, and 29.2% respectively, which was 47.2% in control group. While the combination Huaier and 5-FU could had a stronger inhibiting effect of 20.7% than either single agent alone. As the second step, Transwell cell migration and invasion assays were performed to further identify the motility inhibiting effect induced by different treatment (Fig. 4c, d, e and f). The result indicates that both numbers of migrating and invasive cells were down-regulated by either single agent, and the combination Huaier with 5-FU had a synergistically inhibition on migratory and invasive abilities of Huh28 cells.

Subsequently, the protein expression level of N-cadherin, Vimentin, MMP-2 and MMP-9 associated with metastasis ability of tumor were detected to further determine the effect of different treatments. The results showed that the Huaier combined with 5-FU exerted a stronger inhibiting effect on above protein expression than either single agent (Fig. 4g, h, i, j, k and l).

### Synergetic inhibition of STAT3 activation by combination Huaier and 5-FU treatment

**Fig. 5:**
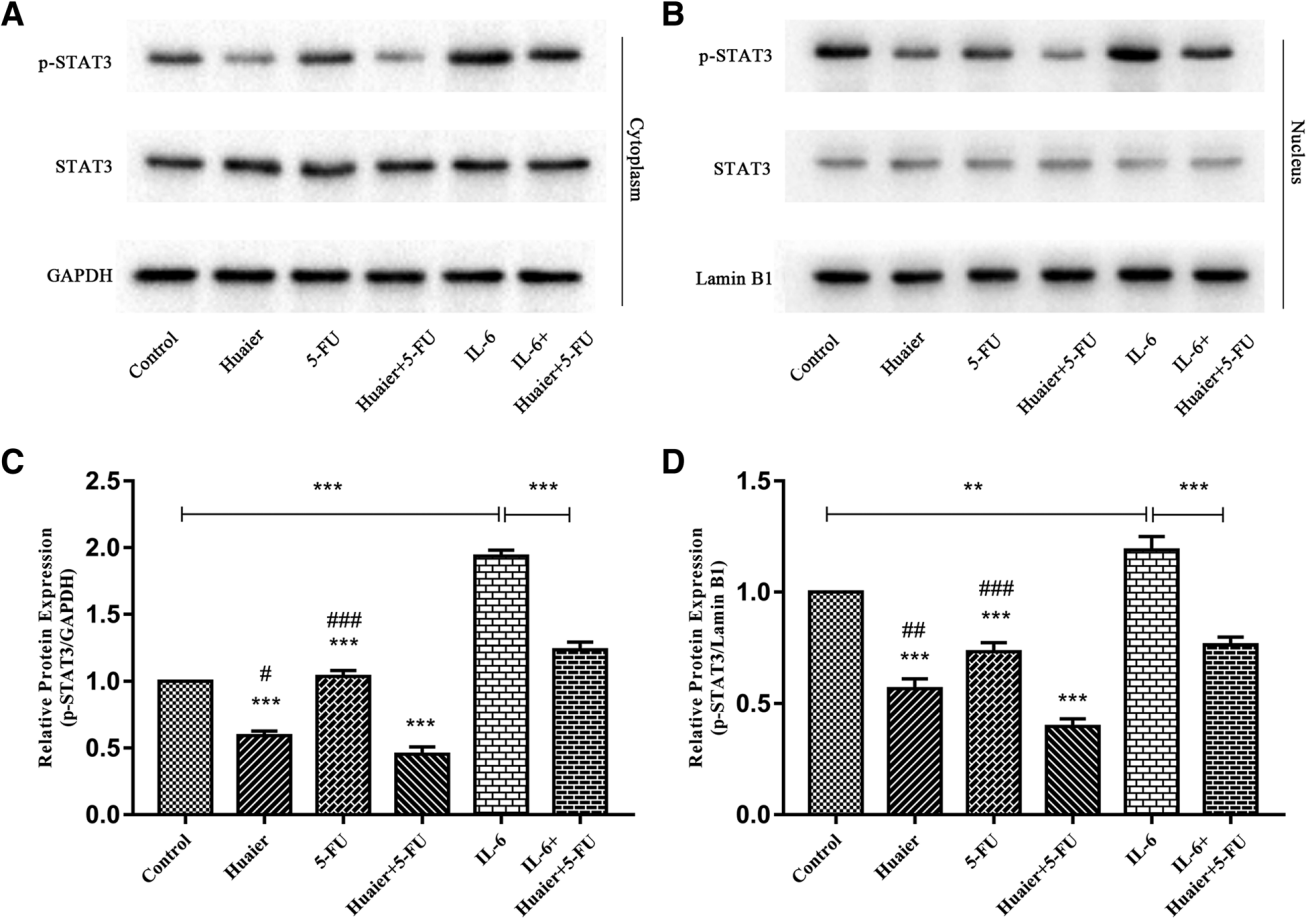
Huaier synergized with 5-FU promoting the inhibition of both constitutive and Inducible STAT3 phosphorylation by IL-6. The total STAT3 and p-STAT proteins expression in cytoplasm (**a** and **b**) and nucleus (**c** and **d**) were detected using Western blot after 48 h treatment with Huaier and 5-FU alone or in combination. In the meantime, the effect of combination therapy on p-STAT3 Induced by IL-6 were also checked. Relative optical density of proteins were analyzed by Image J. All results are expressed as mean ± SD of three independent experiments. ^*^P < 0.05, ^**^P < 0.01, ^***^P < 0.001, compared with control group, while ^#^*P* < 0.05, ^##^*P* < 0.01, ^###^*P* < 0.001, compared with combination group

In view of STAT3 signaling pathway is constitutively activated in cholangiocarcinoma and closely associated with proliferation, apoptosis and metastasis of tumor cells, we then investigated the effect of Huaier and 5-FU alone or their combination on STAT3 and its activation form p-STAT3(tyrosine-phosphorylated STAT3. As shown in Fig. 5a and b, the application of the combined medication could have a strong inhibiting effect of p-STAT3 than either drug alone in cytoplasm of Huh28, while total STAT3 were unimpaired. In addition, a similar reduction of p-STAT3 in nucleus response to different treatments was observed **(**Fig. 5a, b, c and d). To further verify the synergetic inhibition of p-STAT3 by combination Huaier with 5-FU, we used IL-6 to stimulate phosphorylation of STAT3 and checked whether combination Huaier and 5-FU could reverse the effect of IL-6 on p-STAT3. The result shown that a significant rise in P-STAT3 after 48 h incubation with 10 ng/ml IL-6 in both cytoplasm and nucleus of Huh28 cells, while concomitant with Huaier and 5-FU, the p-STAT3 induced by IL-6 were reversed. And then A series of experiments were conducted to verify the effect of Huaier combined with 5-FU on Huh28 cells cultured under the induction of IL-6. The results showed that combination of two drugs could reduce the proliferating effects caused by IL-6, similar results also appeared in Flow cytometry and Transwell cell migration and invasion assays (Additional file 2: Figure S1).

## Discussion

Chemotherapy is one of the comprehensive treatments of cholangiocarcinoma primarily used to slow down the development of tumor and prolong the survival time after surgery [22]. As one of the chemotherapy agents for gastric cancer, breast cancer and colon cancer, 5-FU has also been used as a first-line drug for cholangiocarcinoma. Similar to other chemotherapy drugs, drug resistance and severe side effects during clinical practice are common and limit its usage. Accordingly, researches on the new combination of drugs or chemotherapy sensitizers to improve effectiveness and reduce side effects have become the focus. In recent years, numerous traditional Chinese herb extracts have been used in combination with 5-FU to reduce chemotherapy resistance and enhance anticancer effect. Serval good results in vivo and in vitro have been reported (e.g. curcumin combined with 5-FU in gastric cancer, beta-elemene combined with 5-FU in colon cancer, puerarin combined with 5-FU in esophageal cancer) [23–25]. However, the anticancer effect of the combination Huaier and 5-FU has been less known. Thus, in this study, the effects of Huaier combined with 5-FU on proliferation, apoptosis, cell cycle, invasion and metastasis of cholangiocarcinoma Huh28 cells were systematically assessed, and the mechanism of this combination was explored preliminarily.

Our study verified that Huaier could inhibit the proliferation of cholangiocarcinoma cells in a concentration range of 1.5 to 24 mg/ml. Concentration-dependent and time-dependent manner were also observed. Also, the inhibiting effect of 5-FU on Huh28 cells was investigated using CCK-8 assay. The purpose of combination therapy is to maximize the effectiveness of drug and minimize the side effects [26]. Chou-Talalay Method was adopted to verify whether there exists a synergistic effect of combination therapy experiments between 5-FU with Huaier and choose reasonable dosage [27]. According the median-effect principle, we quantitatively analyzed the combination effects of these two drugs. When 6 mg/ml Huaier combined with 30μg/ml 5-FU, the synergistic effect was the strongest. Furthermore, colony formation assay results confirmed combination therapy effect in another way. Both PCNA and Ki 67 could act as markers of cell proliferation. Thus, the down-regulated expressions of PCNA and Ki 67 after combination therapy further illustrated their inhibiting effect on proliferation.

Apoptosis, also known as programmed cell death, is a fundamental physiological process that is criticalin cell development and tissue homeostasis [28]. Apoptosis regulatory proteins are classified into anti-apoptotic proteins and pro-apoptotic proteins; the loss of balance between these two types of proteins may lead to the generation of tumor [29]. Bcl-2 family (Bcl-xL, Bcl-2, Mcl-1) are vital members of anti-apoptotic proteins that regulate of the apoptotic pathway, and they have been reported to over-express in cholangiocarcinoma [30]. Western blot results show that anti-apoptotic proteins were inhibited after administration, which was consistent with the results of flow cytometry test that the combination of drugs induced apoptosis.

Dysregulation of cell cycle is closely associated with the malignant transformation, and agents may exert anticancer effect through affecting cell cycle [31]. 5-FU is a classical cell cycle-specific antimetabolite that mainly function in S phase [32]. However, Huaier has been reported to produce different cell cycle arrests in different cells [33, 34]. In this study, it was found that combination therapy could induce S phase arrest in cholangiocarcinoma cell lines. Cyclins control cell cycle by activating cyclin-dependent kinase (Cdk) enzymes, and Cyclin A2/CDK2 was primarily active during S phase [35]. In our study, Cyclin A2/ CDK2 was inhibited by Huaier combined 5-FU, which further verified the results of flow cytometry.

The invasion and metastasis of cancer which lead to its poor clinical prognosis are considered important targets for anticancer drug therapy [36]. Epithelial mesenchymal transition (EMT), where epithelial derived tumors acquit mesenchymal stem cells’ properties, indicates the possibility of cancer metastasis, and the up-regulated expression of vimentin and N-cadherin (markers of mesenchymal cells) suggests malignant transformation [37]. Besides, the degradation of extracellular matrix and basement membrane is a critical step in tumor invasion. Matrix metalloproteinases, especially MMP2 and MMP9, are considered the main enzymes capable of degrading extracellular matrix [38]. Huaier has been reported to be able to inhibit the EMT process by suppressing the expressions of N-cadherin and MMP-2 in gastric cancer and breast cancer cell lines [39, 40]. Likewise, a series of experiments performed in this study suggest that Huaier alone or combined with 5-FU could inhibit the invasion and metastasis of cholangiocarcinoma cells and those involved in the down-regulation of N-cadherin, Vimentin, MMP-2 and MMP-9 expression.

STAT3 is sensitive to IL-6 stimulus, and IL-6–activated STAT3 is common in many inflammation-induced tumors [41]. It was observed that patients of Cholangiocarcinoma with high p-STAT3 expression in tissues had poor prognosis, and aberrant stat3 phosphorylation and IL-6/STAT3 signaling were reported in Cholangiocarcinoma cells [42, 43]. Previous studies found that by suppressing STAT3 and p-STAT3 expression in non-small cell lung cancer cell lines, Huaier could inhibit the proliferation and the invasion of tumor [44]. In our study, we found that p-STAT3 expression was down-regulated by Huaier combined with 5-FU, while the expression of STAT3 was not significantly affected. Furthermore, when IL-6 was used to activate the STAT3 pathway, this inhibiting effect was alleviated. Based on the above results, we speculated that combination therapy can down-regulate STAT3 phosphorylation.

## Conclusion

A series of experiments were performed on Huh28 cells in vivo. The result suggests that the combination of Huaier with 5-FU had a synergistic antitumor effect in proliferation, apoptosis, cell cycle and motility of cholangiocarcinoma cells; STAT3 pathway might be a potential target. Huaier would be a complementary therapy for cholangiocarcinoma treatment with promising applications.

## Additional files

Additional file 1: **Table S1.** The fraction affected level (Fa) of Huaier and 5-FU in individual or in combination, and the combination index (CI) values of combination after 48 h. **Table S2.** The dose of Huaier or 5-FU alone to achieve the same fraction affected level of in-combination, and the dose reduction index (DRI) values for Huaier combined with 5-FU after 48 h. (DOCX 18 kb)

Additional file 2: **Figure S1.** Effects of treatment with combination of Huaier and 5-FU in Huh28 cells cultured under the induction of IL-6. The combination of Huaier and 5-FU on colony formation with Huh28 cells induced by IL-6 were shown, and the colony numbers were calculated (A). Flow cytometry histograms of Huh28 cells after different treatments. The percentage of apoptotic cells was calculated (B), and the percentage of cells in different phases was detected (C). Transwell cell migration (D) and invasion (E) assays were performed to further identify the motility inhibiting effect induced by different treatments. All results are expressed as mean ± SD of three independent experiments. ^*^P < 0.05, ^**^P < 0.01, ^***^P < 0.001. (TIF 11095 kb)

## Data Availability

All the data in this research are accessible by connecting with the corresponding author.
