# Supplementary material for: The synergistic antitumor effect of Huaier combined with 5-Florouracil in human cholangiocarcinoma cells
Source: BMC Complement Altern Med. 2019 Aug 7;19:203. doi: 10.1186/s12906-019-2614-5 (PMC6686517; doi:10.1186/s12906-019-2614-5)
Supplement: Supplementary file 1 — Table S1. The fraction affected level (Fa) of Huaier and 5-FU in individual or in combination, and the combination index (CI) values of combination after 48 h. Table S2. The dose of Huaier or 5-FU alone to achieve the same fraction affected level of in-combination, and the dose reduction index (DRI) values for Huaier combined with 5-FU after 48 h. (DOCX 18 kb) [file 12906_2019_2614_MOESM1_ESM.docx]

**Additional file 1: Table S1. The fraction affected level (Fa) of Huaier and 5-FU in individual or in combination, and the combination index(CI) values of combination after 48 h.**

| **Huaier**  **(mg/ml)** | **5-FU**  **(μg/ml)** | **Concentration**  **ratio**  **(Hauier : 5-FU)** | **Fa of**  **Huaier** | **Fa of**  **5-FU** | **Fa of combination** | **CI** |
| --- | --- | --- | --- | --- | --- | --- |
| 1.5 | 7.5 |  | 7.10% | 26.30% | 28.70% | 1.05 |
| 3 | 15 |  | 14.30% | 31.70% | 37.10% | 1.2 |
| 6 | 30 | 1:05 | 35.20% | 43.10% | 71.20% | 0.45 |
| 12 | 60 |  | 56.10% | 51.30% | 82.60% | 0.51 |
| 24 | 120 |  | 82.20% | 59.80% | 91.80% | 0.53 |

CI provides a quantitative measure of the extent of the interaction between two drugs. The CI>1 indicates antagonism; CI<1 indicates synergy and CI=1 indicates additive effects. The CI values<1 indicated that the combination(48h) of Huaier (1.5-24 mg/ml) and 5-FU (7.5-120 μg/ml) was synergistic on Huh28 cells.

**Additional file 1: Table S2. The dose of Huaier or 5-FU alone to achieve the same fraction affected level of in-combination, and the dose reduction index (DRI) values for Huaier combined with 5-FU after 48 h.**

| **Fa of**  **combination** | **Dose of Huaier(mg/ml)** | **Dose of**  **5-FU(μg/ml)** | **DRI of**  **Huaier** | **DRI of**  **5-FU** |
| --- | --- | --- | --- | --- |
| 28.7% | 5.00 | 9.96 | 3.33 | 1.33 |
| 37.1% | 6.47 | 20.47 | 2.16 | 1.36 |
| 71.2% | 17.07 | 305.55 | 2.85 | 10.19 |
| 82.6% | 26.55 | 1046.02 | 2.21 | 17.43 |
| 91.8% | 47.46 | 5276.55 | 1.98 | 43.97 |

The DRI indicates the fold of the dose reduction of tested Huaier and 5-FU in-combination compared with that of each individual drug. To achieve a 71.2% of inhibition effect in Huh28 cells proliferation when the CI was at the lowest (0.45, as seen in Table1A) in combination after 48 h, the concentrations of Huaier and 5-FU were reduced 2.85~ folds and 10.19~folds in combination while the dose of them reached to 17.07 mg/ml and 305.55 μg/ml in individual.
