# Supplementary figures and images for: The synergistic antitumor effect of Huaier combined with 5-Florouracil in human cholangiocarcinoma cells
Source: BMC Complement Altern Med. 2019 Aug 7;19:203. doi: 10.1186/s12906-019-2614-5 (PMC6686517; doi:10.1186/s12906-019-2614-5)

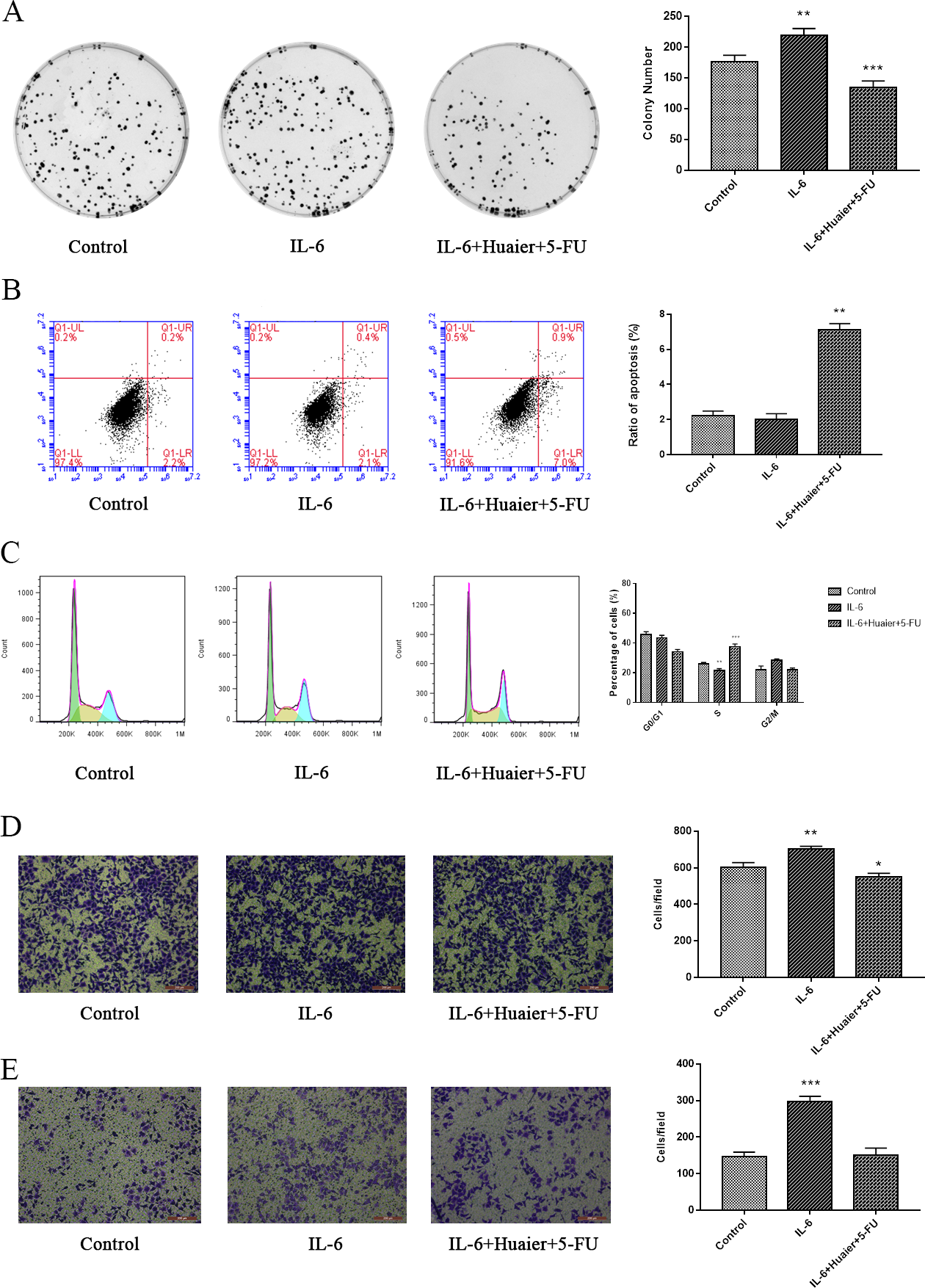

Supplement: Supplementary file 2 — Figure S1. Effects of treatment with combination of Huaier and 5-FU in Huh28 cells cultured under the induction of IL-6. The combination of Huaier and 5-FU on colony formation with Huh28 cells induced by IL-6 were shown, and the colony numbers were calculated (A). Flow cytometry histograms of Huh28 cells after different treatments. The percentage of apoptotic cells was calculated (B), and the percentage of cells in different phases was detected (C). Transwell cell migration (D) and invasion (E) assays were performed to further identify the motility inhibiting effect induced by different treatments. All results are expressed as mean ± SD of three independent experiments. *P < 0.05, **P < 0.01, ***P < 0.001. (TIF 11095 kb) [file 12906_2019_2614_MOESM2_ESM.tif]
